# Integrated microRNA and messenger RNA analysis in aortic stenosis

**DOI:** 10.1038/srep36904

**Published:** 2016-11-23

**Authors:** Sean Coffey, Michael J. A. Williams, L. Vicky Phillips, Ivor F. Galvin, Richard W. Bunton, Gregory T. Jones

**Affiliations:** 1 Kolling Institute, University of Sydney, Australia; 2 Department of Cardiology, Royal North Shore Hospital, Sydney, Australia; 3 Department of Medicine, Dunedin School of Medicine, University of Otago, Dunedin, New Zealand; 4 Department of Surgical Sciences, Dunedin School of Medicine, University of Otago, Dunedin, New Zealand

## Abstract

Aortic valve stenosis (AS) is a major cause of morbidity and mortality, with no effective medical therapies. Investigation into the underlying biology of AS in humans is limited by difficulties in obtaining healthy valvular tissue for use as a control group. However, micro-ribonucleic acids (miRNAs) are stable in post-mortem tissue. We compared valve specimens from patients undergoing aortic valve replacement for AS to non-diseased cadaveric valves. We found 106 differentially expressed miRNAs (p < 0.05, adjusted for multiple comparisons) on microarray analysis, with highly correlated expression among up- and down-regulated miRNAs. Integrated miRNA/gene expression analysis validated the microarray results as a whole, while quantitative polymerase chain reaction confirmed downregulation of miR-122-5p, miR-625-5p, miR-30e-5p and upregulation of miR-21-5p and miR-221-3p. Pathway analysis of the integrated miRNA/mRNA network identified pathways predominantly involved in extracellular matrix function. A number of currently available therapies target products of upregulated genes in the integrated miRNA/mRNA network, with these genes being predominantly more peripheral members of the network. The identification of a group of tissue miRNA associated with AS may contribute to the development of new therapeutic approaches to AS. This study highlights the importance of systems biology-based approaches to complex diseases.

Calcific aortic valve disease (CAVD) is an increasingly important disorder due to the aging of the population^1^,^2^. End-stage CAVD results in severe aortic stenosis (AS), a condition where trials of medical therapies have been largely disappointing^3^,^4^,^5^, and the only effective treatment is replacement of the valve. Aortic valve replacement (AVR), either surgical or trans-catheter, is however associated with substantial morbidity and mortality, especially in older age groups. While surgical results in younger age groups are excellent, it is possible that with increasing life expectancy, these patients will require further cardiac surgery at a later age, with the first operation making subsequent procedures more hazardous. Therefore, there is a pressing need for development of medical therapies to slow the progression of the disease in all age groups.

The lack of suitable controls has made molecular investigations into human valvular diseases difficult. Micro-ribonucleic acids (microRNAs/miRNAs) are small, non-coding RNAs that act to inhibit translation of messenger RNA (mRNA) into proteins and are involved in many cardiovascular diseases^6^. MiRNAs have been well reported as being stable in post-mortem tissue under a variety of conditions, most likely due to their small size and binding to proteins such as Argonaute-2^7^,^8^. Previous studies involving miRNAs in CAVD have used microarrays to compare stenotic to regurgitant bicuspid valves^9^, tricuspid to bicuspid valves^10^, and endothelial cells in varying flow conditions^11^, as well as manipulation of a single miRNA, miR-30b, in aortic valvular interstitial cells^12^. However, only limited numbers of tricuspid aortic valves have been analyzed with whole miRNome coverage^13^.

In this study we applied a systems biology approach to identify potential therapeutic targets in AS. To do this, we examined valve miRNA profiles in patients with and without AS. We validated these findings using miRNA quantitative polymerase chain reaction (qPCR) and a novel whole-transcriptome mRNA approach. Finally, we used the combined miRNA/mRNA network for pathway analysis and to identify potential targets for treatment, looking in particular for drugs suitable for repurposing as AS disease modifying agents. This information should be useful both as a description of miRNA expression networks in AS and as a platform for further experiments to move these findings closer to human use.

## Results

We compared aortic valve samples from participants with AS undergoing AVR to samples obtained post-mortem. On average control participants were significantly younger than participants with AS (Table 1). As would be expected in those undergoing AVR, patients with AS had severe disease, with a mean aortic valve maximum velocity of 4.4 m/s, standard deviation (sd) 0.6 m/s, mean pressure gradient 49 mmHg (sd 13 mmHg), and calculated aortic valve area 0.8 cm^2^ (sd 0.2 cm^2^).

Total RNA yield from post-mortem tissue was higher than from surgically explanted tissue (153 ng/μl and 101 ng/μl respectively) but of a similar level of purity (mean 260:280 ratio after reverse transcription 2.05 (range 2.02–2.1) and 2.01 (range 1.90–2.05) in post-mortem tissue and surgically explanted valves respectively). Levels of the non-coding RNA SNORD44 were similar between groups (threshold cycle (Ct) values 22.1 (sd 5.6) in the AS group (n = 16) compared to 21.7 (sd 4.3) in the control group (n = 36), t-test p-value 0.81), while exogenous spike-in control cel-miR-39 levels were only minimally different (mean Ct 20.3 (sd 0.8) in AS group compared to 19.8 (sd 0.9) in control group, t-test p-value 0.03).

### Differentially expressed microRNA profiles in diseased aortic valve tissue

**Figure 1:**
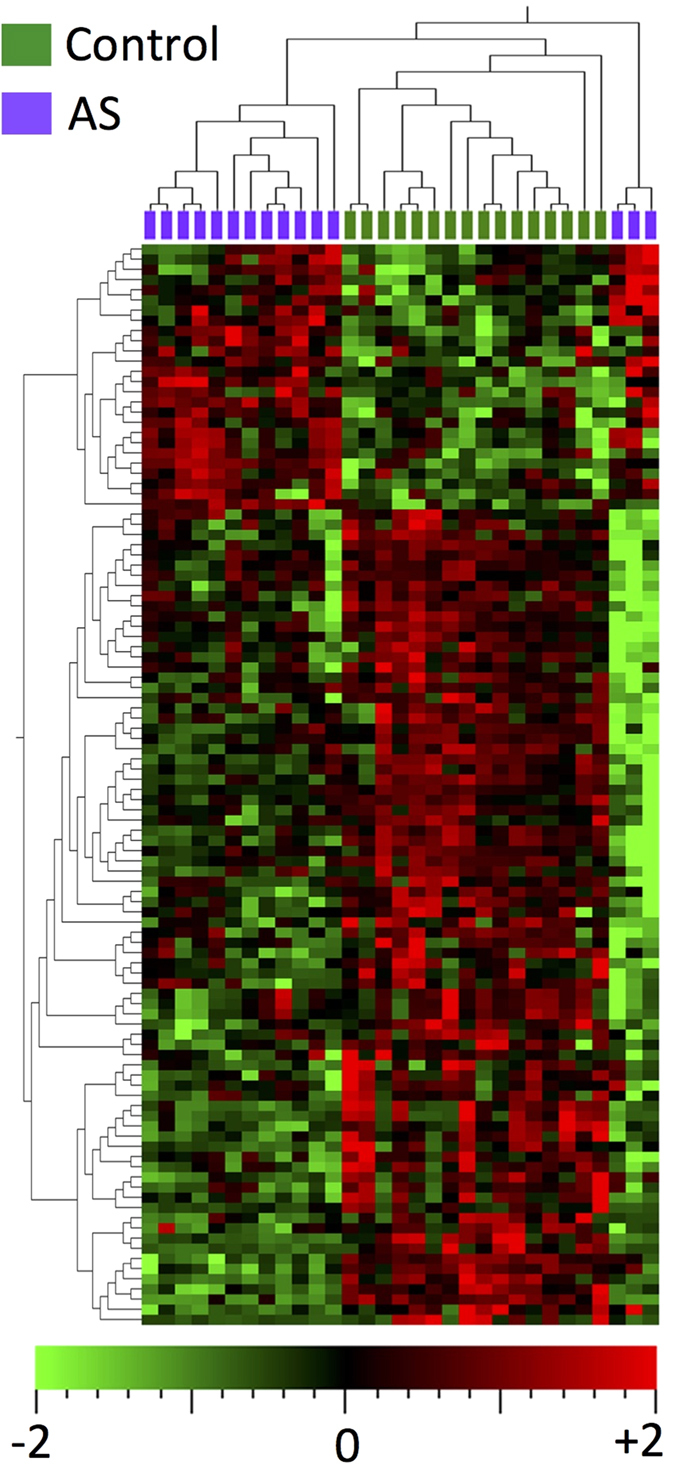
Heatmap of differentially expressed miRNAs in aortic stenosis (adjusted p-value <0.05). Hierarchical clustering shows clear grouping according to presence or absence of aortic stenosis. The aortic stenosis samples to the right of the figure are outliers on the principal components analysis plot (Supplementary Figure 1 and Supplemental movie), but still cluster with other aortic stenosis samples on the first principal component. The miRNAs are listed in Supplementary Table 1.

Whole miRNome profiles of 15 severely diseased aortic valve leaflets were compared to 16 control valves. After quality filtering and normalization using robust multi-array averaging, 106 miRNAs were differentially expressed between the AS and control groups at a multiple testing adjusted p-value <0.05 (Supplementary Table S1). The majority (80/106) of miRNAs were down-regulated in stenotic aortic valve tissue. Using these differentially expressed miRNAs, the miRNA profile clearly distinguished those with and without AS using hierarchical clustering (Fig. 1) and principal components analysis (Supplementary Fig. S1).

**Figure 2:**
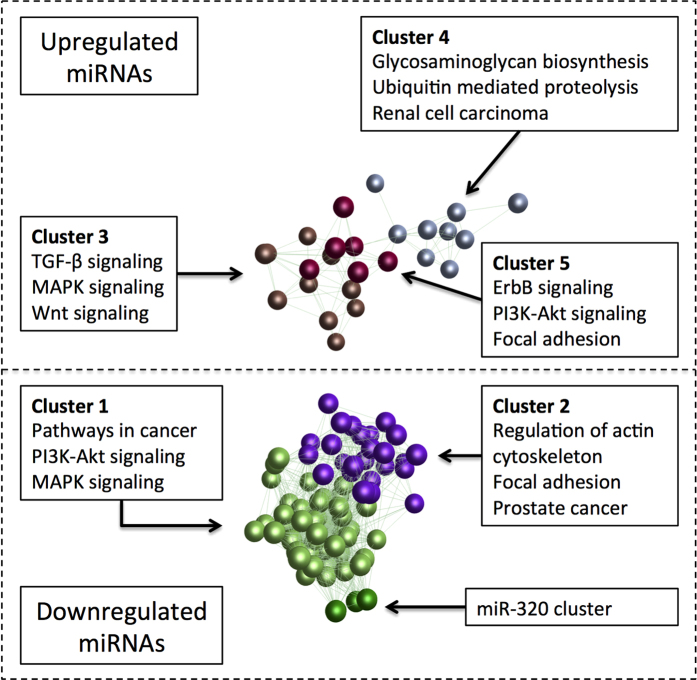
Co-expression network of differentially expressed miRNAs in aortic stenosis. Differentially expressed miRNAs (adjusted p < 0.05) in severely diseased aortic valve tissue with Pearson correlation coefficient more than 50%. Different colours distinguish the different groups identified by the Markov clustering algorithm. Boxes list the top three KEGG pathways associated with the miRNAs in each cluster by DIANA miRPath. The dashed separator is used to separate up- and down-regulated miRNAs, but the network was analysed as a whole. Abbreviations: KEGG, Kyoto Encyclopedia of Genes and Genomes; MAPK, mitogen-activated protein kinase; PI3K, phosphatidylinositol-4,5-bisphosphate 3-kinase; TGF, transforming growth factor.

Most miRNAs formed correlated clusters of expression (Supplementary Figure S2). The majority of down-regulated miRNAs showed highly correlated expression (Pearson correlation coefficient ≥70%), with the most suppressed, miR-122-5p, at the core of the miRNA network. Up-regulated miRNAs were correlated to a lesser degree, but the majority were still correlated at a Pearson correlation coefficient ≥50%, and had miR-21-5p at the core of the major network. Network analysis with the Markov clustering algorithm showed five separate clusters of co-expressed miRNAs as well as a cluster consisting of miR-320a, -320b, and -320c (Fig. 2). Pathway analysis of the clusters showed that although there was considerable overlap, in particular with the MAPK and Pl3K-Akt signalling pathways, the clusters had a distinct set of associated pathways (Table 2), and cluster 3, which contained miR-21-5p, miR-221-3p and miR-222-3p, was most associated with TGF-β signalling (adjusted p-value 1.2 × 10^−16^).

**Figure 3:**
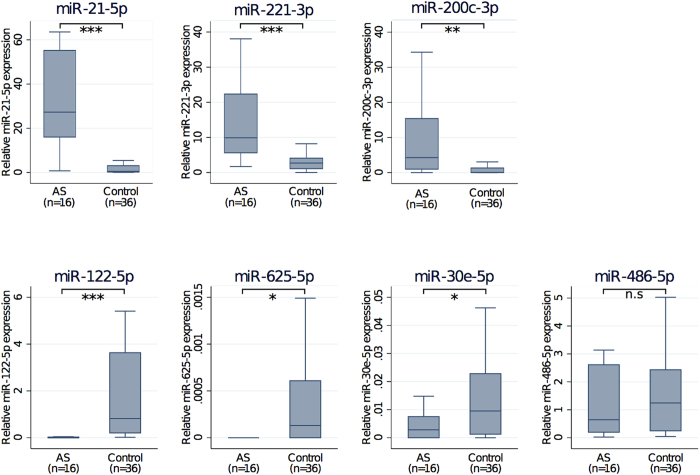
microRNA expression in aortic valve tissue as measured by quantitative polymerase chain reaction. Abbreviations: n.s., not significant. Symbols: *p < 0.05; **p < 0.01; ***p < 0.0001.

To validate specific miRNAs, qPCR was used to examine 16 severely diseased samples and 36 control samples (including the original 15 severely diseased samples and 16 control samples) (Fig. 3). Comparing the AS to control groups, we confirmed differences in levels of miR-122-5p (Mann-Whitney p < 0.0001), miR-21-5p (p < 0.0001), miR-625-5p (p = 0.017), miR-221-3p (p < 0.0001) and miR-30e-5p (p = 0.012). miR-200c-3p levels were statistically different (p = 0.001), but in the opposite direction to the microarray results (up-regulated in diseased AS samples by qPCR analysis), while validation qPCR of miR-486-5p showed no difference between the two groups (p = 0.74).

### Integrated miRNA/mRNA analysis

There were 3297 mRNAs differentially expressed between severely diseased and control aortic valve tissue (adjusted p-value <0.05), 3292 of which were recognized by the miRNA/mRNA integrated analysis software MAGIA^2^. When paired with differentially expressed miRNA levels from the same sample, MAGIA^2^ identified 3707 statistically significant miRNA-mRNA interactions, higher than any random permutation (p-value <0.01, 95% confidence interval 0–0.036).

ClueGO identified ten KEGG pathways associated with this network (Table 3), with the three most significant being focal adhesion, regulation of actin cytoskeleton, and extracellular matrix receptor interaction. By comparison, only three KEGG pathways were identified when using the differentially expressed mRNAs alone (Table 3).

### Network based drug discovery

**Figure 4:**
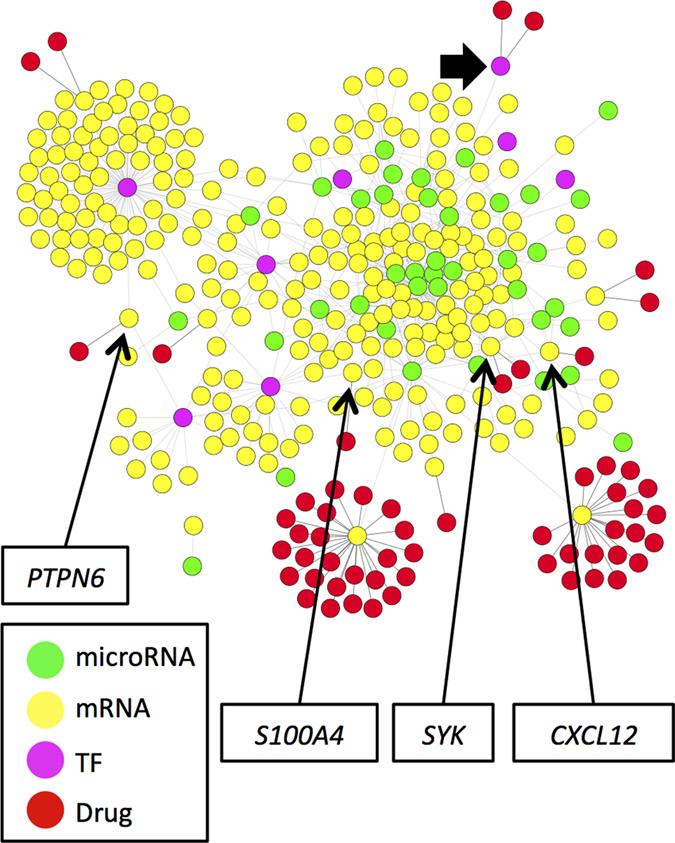
The aortic stenosis associated miRNA/upregulated mRNA subnetwork. Only mRNA upregulated more than two-fold and their immediate neighbours are shown. Labels indicate genes with two or more connections in the full miRNA/mRNA network (not shown), with the drugs that target them listed in Table 2. For comparison, transforming growth factor beta 1 (marked by the filled arrow), despite being 3.6 fold increased in severely diseased valves, has only one connection to other members of the full network. Abbreviations: *CXCL12*, stromal cell-derived factor 1; *PTPN6*, Tyrosine-protein phosphatase non-receptor type 6; *S100A4*, S100 calcium binding protein A4; *SYK*, spleen tyrosine kinase; TF, transcription factor.

For target discovery, we focused on upregulated genes in the integrated miRNA/mRNA network with fold change of two or greater. A total of 57 potential drug-gene interactions were identified in the DGIdb database that might reduce the levels of the corresponding gene products (Fig. 4). The majority of these genes are on the periphery of the network. There were only four targeted genes with two or more connections to other parts of the network (Table 4). Spleen tyrosine kinase (*SYK*) was the most connected targeted mRNA, and transforming growth factor (*TGF*)-β, while not being highly connected in the network, was also identified as a drug target.

## Discussion

In this study we have shown that there is a markedly different miRNA profile in the aortic valve tissue of patients with and without aortic stenosis. We used a novel approach based on the relationship between miRNAs and their mRNA targets to validate the miRNA microarray results as a whole. Integration of miRNA and mRNA profiles shows that pathways involved in extracellular matrix function are the most significant in aortic stenosis. In addition, we have shown pathway analysis based on integrated miRNA/mRNA profiles is more sensitive than pathway analysis based on mRNA profiles alone. Finally, we have shown that currently available pharmacotherapies exist that could be investigated for use in AS by targeting members of the miRNA/mRNA network.

Two specific miRNAs stand out as being of particular importance in the pathobiology of AS. miR-122-5p expression was almost completely suppressed in severely diseased aortic valves. The finding of a role of miR-122-5p in CAVD was unexpected, as miR-122-5p is primarily expressed in the liver, with most attention focusing on its role in hepatitis C infection. miR-122-5p has the distinction of being the first miR to be manipulated in human clinical trials, where an antagomiR, miravirsen, has shown promising early results^14^. It is, however, worth noting that increasing the levels of a particular miR, which would be required if miR-122-5p were pursued as a therapeutic target in AS, is generally viewed as more challenging than suppressing that miR.

miR-122-5p is also involved in lipid metabolism, and this is a possible reason for the differential expression in CAVD. miR-122-5p tends to inhibit fatty acid oxidation, and promotes fatty acid and triglyceride biosynthesis^15^. A trial of anti-miR-122 in chimpanzees (designed primarily to assess the effect on hepatitis C virus) showed a 25 to 54% reduction in low-density lipoprotein, and a 23 to 42% reduction in apolipoprotein apo-B^16^. In addition to its involvement in lipid metabolism, a recent study has also shown that miR-122-5p directly targets *TGF-β* and is downregulated in the myocardium of AS patients with more extensive myocardial fibrosis^17^. Interestingly, hypothesis-free pathway analysis of the aortic valvular transcriptome performed by Bossé *et al.* showed that the most significantly disease-associated pathway was hepatic fibrosis/hepatic stellate cell activation^18^. At the time there was no obvious association between this pathway and underlying mechanisms of CAVD, but our current observation may suggest that miR-122-5p provides this link. Of note, miR-122-5p deletion in mice leads to hepatic fibrosis^19^, while overexpression leads to suppression of stellate cell proliferation and reduced collagen production^20^, and, conversely, stellate cell activation leads to downregulation of miR-122-5p^21^. Corresponding biological processes may be at work in diseased aortic valves, where the aortic valve interstitial cell acts locally in a similar way to that of the hepatic stellate cell in the liver.

In contrast to miR-122-5p, miR-21-5p expression showed significant upregulated expression in severely diseased aortic valves. miR-21-5p has been strongly associated with both cardiac and extra cardiac fibrotic processes^22^,^23^,^24^. Its expression can be induced by TGF-β, via both direct and indirect (pressure overload) mechanisms, in experimental systems^25^. Given the previously well-described increase in TGF- β signalling in advanced CAVD^26^, an observation replicated in this current study, we suggest that TGF-β overexpression may well be the cause of the increased miR-21-5p activity observed in diseased aortic valves. In addition, miR-21-5p expression is induced by states of low shear stress^27^, which is the predominant flow pattern experienced by the fibrosa surface of the aortic valve, and which is the site of most intensive CAVD activity^28^. Our finding of increased miR-21-5p in CAVD is therefore consistent with the previous literature.

By combining miRNA results with gene expression information, we have shown that the number of interactions between differentially expressed miRNAs and mRNAs is much higher than obtained by chance. In addition, these interactions allow refinement of the pathways involved in aortic stenosis, identifying more statistically significant pathways. Components of the pathways identified have previously been implicated in aortic stenosis^26^,^29^,^30^, providing useful external validation of our findings. Moreover, given miRNA involvement in many of the pathways identified as central to the disease, this suggests that miRNAs may well serve as potential therapeutic targets in their own right. The relatively small number of upregulated genes identified as being targets for current drugs, and especially the very low number of highly connected genes, means that novel therapies are likely to be important in treating AS. Based on analysis of the miRNA/mRNA network, a number of therapies, such as those targeting *SYK* and *TGF-β*, could be potentially repurposed for treatment of AS. While the drugs identified have significant side effect profiles, which may limit their use over the relatively long time periods that are likely to be necessary for AS disease modification, active development of many of the identified drugs, as well as others not yet listed in the database, is ongoing^31^.

A limitation of this study is the potential for systematic differences in RNA levels between aortic valves obtained post-mortem and explanted during surgery. To overcome this, we focused primarily on miRNA, which maintains stable signal even in degraded human tissue RNA preparations^32^ and has been shown to undergo few signs of degradation in the first 24 hours post-mortem^7^,^33^,^34^. Although we applied a cautious approach to our mRNA analysis, using it as a secondary validation of our miRNA findings, it is nevertheless worth noting that we found minimal differences between groups in terms of RNA purity or levels of endogenous (SNORD44) mRNA with RNA yield actually being higher in cadaveric samples.

The lack of clinical information about the control aortic valves is also a potential limitation, as we were unable to exclude those with systemic diseases potentially contributing to the CAVD process, such as end stage renal disease, or account for differences in medication between groups. Another limitation is the difference in ages between cases and controls. The limited availability of tissue samples meant that we were unable to pre-select specific age ranges. We conducted a separate analysis into the possible effects of age on miRNA expression, which is detailed in the Supplementary Material (Supplementary Figure 3), and found that age has minimal, if any, effects on those miRNAs assessed by qPCR. Finally, the overall sample size, although matching our power calculations prior to study commencement, is small. Through robust quality control measures, controlling for multiple statistical testing and using two separate methods to validate the miRNA results, we are likely to have a low false discovery rate. However, we may not have identified miRNAs or mRNAs with low levels of expression or with small differences between groups that are nevertheless important in the disease process.

Before translating these results into clinical use, a number of hurdles must be overcome. Ideally manipulation of drug targets in preclinical models, moving from *in-vitro* to *in-vivo* systems, would allow testing of our results in a robust fashion. However, development of new therapies in AS has been hindered by the lack of accurate disease models, with the failure of the statin trials following promising results in both *in-vitro* systems and *in-vivo* animal models^35^,^36^. Alternative methods to investigate potential drug targets in humans include using observational techniques such as Mendelian randomization^37^, or using advanced surrogate markers of disease activity^38^. Additional studies are needed, using techniques such as these, in conjunction with more advanced preclinical experimental systems^39^, to clarify the effect of manipulating the identified miRNAs on the AS disease process.

In conclusion, this study identified markedly different miRNA profiles in aortic valves with advanced CAVD compared to controls. Pathway and network analysis of the integrated miRNA/mRNA network appeared consistent with previous knowledge of the disease process, as well as raising new avenues for exploration. These findings can contribute to the development of new therapeutic approaches to AS, and highlight the importance of systems biology based approaches to complex diseases.

## Methods

### Case and control selection

We enrolled participants with tricuspid aortic valves and moderate to severe aortic stenosis^40^, based on transthoracic echocardiographic findings of: maximum aortic velocity ≥3.0 m/s, peak gradient ≥35 mmHg, mean gradient ≥20 mmHg, or calculated aortic valve area ≤1.5 cm^2^. In addition, subjects were required to have normal left ventricular systolic function, defined as an echocardiographic biplane ejection fraction of 50% or higher, and stable clinical features.

We excluded participants with bicuspid aortic valve (BAV) due to the additional complexities of the different BAV phenotypes, and excluded patients with other congenital cardiac abnormalities or other significant structural heart diseases, such as valvular regurgitation of more than mild severity. Subjects with a past history of rheumatic heart disease or current major systemic disease (such as advanced chronic kidney disease) were also excluded.

Control aortic valve tissue was obtained from post-mortem specimens. To preserve anonymity of the deceased, the only clinical information provided to the research team was age and sex. All methods were performed according to the ethical guidelines of the 1975 Declaration of Helsinki, and the protocol was approved by the New Zealand Multi-region and Lower South ethics committees (LRS/11/07/030), with written informed consent being obtained from all participants or next-of-kin.

### Sample processing

Aortic valve tissue from subjects with AS was obtained at the time of valve replacement surgery, and placed in saline immediately after explantation from the patient. The valve tissue was dissected into representative samples and processed in RNAlater^®^ (Life Technologies, Carlsbad, CA, USA). The most severely diseased part of the leaflet was used for subsequent analysis (Supplementary Figure S3).

Control aortic valve tissue was removed *en bloc* at the time of post-mortem, which was within 24 hours after time of death. All three valve leaflets were carefully grossly examined for evidence of sclerotic disease and only valves without evidence of calcific disease and consistent morphology in all three leaflets were selected. A representative leaflet was processed for histology, while the remaining two were processed in RNAlater^®^.

### Micro and messenger RNA microarray analysis

Total RNA, including miRNA, was extracted using microRNA purification kits (Norgen Biotek, Thorold, Canada) according to the manufacturer’s instructions. To ensure similar RNA extraction between surgical and cadaveric samples, we assessed total RNA yield, 260:280 ratio, and levels of the non-coding RNA SNORD44 and the spike-in *C. elegans* cel-miR-39, as measured by qPCR threshold cycle values. MicroRNA was analysed using Affymetrix miRNA v2.0 GeneChips, while mRNA was analysed using PrimeView Human gene expression GeneChips (Affymetrix, Santa Clara, California). Both array platforms were used according to manufacturers instructions and run in an accredited service laboratory (Otago Genomics & Bioinformatics Facility, University of Otago, Dunedin, NZ).

Whole miRnome and mRNA results were normalised using Robust Multi-array Averaging and analyzed using principal components analysis (PCA) and unsupervised hierarchical clustering (Qlucore Omics Explorer version 2.3, Qlucore AB, Lund, Sweden). The mRNA probes were collapsed to unique Ensembl identifiers. To reduce the complexity of the analysis, we focused on miRNA and mRNA with differential expression between groups, taking a Benjamini-Hochberg adjusted p-value (false discovery rate) of less than 0.05 to be statistically significant. When few individual markers were differentially expressed at this level between the groups but a clear group structure could be determined using PCA and/or heatmap, we used markers with p-values lower than the highest level allowing visual differentiation, as long as the unadjusted p-value was less than 0.05.

### miRNA network and pathway analysis

Differentially expressed miRNAs with Pearson correlation coefficient greater than 0.5 were loaded into BioLayout Express 3D^41^. We then ran the Markov Clustering (MCL) algorithm^42^ to examine clusters of co-expressing miRNAs in the network. The inflation parameter, which adjusts the granularity of the clusters was set to 2.0, a default setting. After this network analysis, we inputted the miRNAs from each cluster individually into DIANA miRPath v2.0, using the DIANA microT-CDS algorithm with the default threshold of 0.8 for target identification^43^.

### Quantitative polymerase chain reaction analysis of miRNAs

SYBR^®^ Green polymerase chain reaction probes (Quanta Biosciences Inc, Gaithersburg, USA) were used for qPCR confirmation of specific miRNAs. Any probe assay with a cycle threshold (C_T_) over 35 was defined as not detected. Expression levels were normalised using the delta C_T_ method^44^, referenced to two endogenous miRNA normalisation controls, miR-16-5p (based on previous use) and miR-151-5p (based on the Normfinder stability score of tissue microarray data)^45^,^46^. Statistical analysis of qPCR results was conducted using Stata/SE v12.1 (StataCorp, College Station, USA), with p < 0.05 considered to be significant. As the qPCR distributions were not normally distributed, we used nonparametric tests (for continuous variables: Mann-Whitney test for two-group comparisons; for categorical variables: chi-squared test). To show the main body of data without log-transformation, figures with qPCR data had extreme outliers removed but all qPCR data was used for statistical testing.

### Integrated miRNA/mRNA analysis

We integrated the miRNA profiles with mRNA profiles in 10 samples each from cases and controls. The differentially expressed mRNA and miRNA profiles of these 20 samples were integrated without further filtering on a per-sample basis using MAGIA^2^, where we used Spearman correlation and the mean DIANA microT target score to map the miRNAs to potential targets^47^. To test the statistical significance of the overall miRNA microarray results, the number of statistically significant (Benjamini-Hochberg adjusted p-value <0.05) interactions identified by MAGIA^2^ was then used for calculation of a p-value based on 100 random permutations of expressed miRNA labels, with the 95% confidence interval taken from the binomial distribution.

For pathway analysis using mRNA profiles, the statistically significant interactions were loaded into Cytoscape 3.0.0, and significant KEGG pathways identified using ClueGO v2.1.4, where we looked for enrichment or depletion using the two-sided hypergeometric test (Benjamini-Hochberg adjusted p-value <0.05)^48^,^49^. To identify drugs that might modulate the disease process, we focused on mRNA upregulated more than 2-fold in the miRNA/mRNA integrated network (all of which are differentially expressed with adjusted p-value <0.05). This list was entered into DGIdb^50^, and drugs listed as inhibitor, antagonist or antibody were identified. To examine whether these drugs acted on central nodes in the network, analysis of the entire miRNA/mRNA network using the Network Analyzer tool version 2.7 was performed, focusing on genes with network degree more than two (those with one degree consisted 38% of the nodes in the network)^51^.

### Data Availability

The data sets supporting the results of this article are available in the ArrayExpress database (www.ebi.ac.uk/arrayexpress) under accession numbers E-MTAB-2766 and E-MTAB-3105.

## Additional Information

**Accession codes:** The data sets supporting the results of this article are available in the ArrayExpress database (www.ebi.ac.uk/arrayexpress) under accession numbers E-MTAB-2766 and E-MTAB-3105.

**How to cite this article**: Coffey, S. *et al.* Integrated microRNA and messenger RNA analysis in aortic stenosis. *Sci. Rep.* **6**, 36904; doi: 10.1038/srep36904 (2016).

**Publisher’s note:** Springer Nature remains neutral with regard to jurisdictional claims in published maps and institutional affiliations.

## Supplementary Material

Supplementary Information

## Figures and Tables

**Table 1:** Demographics of participants.

	Controls	Aortic stenosis	p-value
A. Microarray	n = 16	n = 15	
Age, mean (sd)	55.3 (14.4)	78.0 (5.8)	<0.001
Male gender, n (%)	12 (75%)	9 (60%)	0.46
B. qPCR validation	n = 36	n = 16	
Age, mean (sd)	59.4 (17.9)	78.6 (6.0)	<0.001
Male gender, n (%)	25 (69%)	11 (69%)	1.0

Samples used for (A) microarray and (B) qPCR analysis. Only the demographics for those samples passing quality control are included. Age was compared between aortic stenosis and control groups using the t-test, while gender was compared using Fisher’s exact test. Abbreviations: n, number; qPCR, quantitative polymerase chain reaction; sd, standard deviation.

**Table 2:** Pathway analysis of clusters identified on analysis of miRNA co-expression network.

Example miRNAs	KEGG pathway	p-value	Number of miRNA gene targets in pathway	Number of miRNAs involved in pathway
**Cluster 1**				
miR-200c-3p, -30d-5p, -30e-5p, -422	Pathways in cancer	1.6E-42	194	46
	PI3K-Akt signalling pathway	2.3E-37	184	44
	MAPK signalling pathway	3.1E-32	155	47
**Cluster 2**				
miR-92a-3p,-122-5p, -133a/b, -486-5p	Regulation of actin cytoskeleton	2.6E-39	105	22
	Focal adhesion	9.5E-37	98	24
	Prostate cancer	4.4E-24	49	21
**Cluster 3**				
miR-21-5p, -221-3p, -222-3p	TGF-β signalling pathway	1.2E-16	31	10
	MAPK signalling pathway	2.7E-10	66	9
	Wnt signalling pathway	1.0E-09	44	10
**Cluster 4**				
miR-3197	Glycosaminoglycan biosynthesis	7.0E-14	5	3
	Ubiquitin mediated proteolysis	5.8E-10	32	4
	Renal cell carcinoma	1.2E-07	19	5
**Cluster 5**				
let-7f-5p, -7i-5p, miR-27b-3p	ErbB signalling pathway	8.3E-13	28	6
	PI3K-Akt signalling pathway	1.7E-12	72	6
	Focal adhesion	7.6E-11	47	6

This analysis used differentially expressed miRNAs (adjusted p < 0.05) in severely diseased aortic valve tissue with Pearson correlation coefficient more than 50%. The clusters can be seen in Fig. 2. Representative miRNAs for each cluster are shown. Only the top three KEGG pathways associated with the miRNAs in each cluster by DIANA miRPath are shown. Abbreviations: KEGG, Kyoto Encyclopedia of Genes and Genomes; MAPK, mitogen-activated protein kinase; PI3K, phosphatidylinositol-4, 5-bisphosphate 3-kinase; TGF, transforming growth factor.

**Table 3:** Pathway analysis incorporating mRNA profiles.

	KEGG pathway	Adjusted p-value
A. Integrated miRNA/mRNA analysis	Focal adhesion	0.0001
	Regulation of actin cytoskeleton	0.002
	ECM-receptor interaction	0.002
	Dorso-ventral axis formation	0.003
	PI3K-Akt signaling pathway	0.003
	Platelet activation	0.008
	Pathways in cancer	0.015
	ErbB signaling pathway	0.045
	Tight junction	0.046
	Leukocyte transendothelial migration	0.047
B. mRNA analysis	Focal adhesion	0.001
	Olfactory transduction	0.006
	PI3K-Akt signaling pathway	0.034

Pathways were identified using ClueGO in Cytoscape for (A) integrated miRNA/mRNA results and (B) mRNA results only, using differentially expressed miRNAs and genes. Abbreviations: ECM, extracellular matrix; KEGG, Kyoto Encyclopedia of Genes and Genomes; PI3K, phosphatidylinositol-4, 5-bisphosphate 3-kinase.

**Table 4:** Lists of identified drugs targeting upregulated mRNAs with fold change and network degree both greater than two.

mRNA	Symbol	Fold change	Adjusted p-value	Network degree	Drugs
Spleen tyrosine kinase	*SYK*	2.2	0.004	7	Fostamatinib
					R343
Stromal cell-derived factor 1	*CXCL12*	3.8	0.014	3	Tinzaparin
Tyrosine-protein phosphatase non-receptor type 6	*PTPN6*	2.2	0.007	2	Sodium stibogluconate
S100 calcium binding protein A4	*S100A4*	2.2	0.006	2	Trifluoperazine
Transforming growth factor, beta 1	*TGFB1*	3.6	0.028	1	Hyaluronidase Fresolimumab

Transforming growth factor, beta 1 is also included for comparison. The network degree refers to number of connections in the full mRNA/microRNA network. The adjusted p-value is the Benjamini-Hochberg adjusted p-value for the t-test for differential mRNA expression between diseased and control aortic valves.
