# Supplementary material for: Integrated microRNA and messenger RNA analysis in aortic stenosis
Source: Sci Rep. 2016 Nov 23;6:36904. doi: 10.1038/srep36904 (PMC5120312; doi:10.1038/srep36904)
Supplement: Supplementary Information [file srep36904-s1.pdf]

Supplementary material for “Integrated microRNA and messenger RNA analysis in aortic stenosis.”

Sean Coffey, Michael J.A. Williams, L Vicky Phillips, Ivor F Galvin, Richard W

Bunton, Gregory T Jones

Supplementary analysis

Interaction between age and miRNA expression

Background
The controls we used were not matched by age to our participants with aortic stenosis. We therefore wished to examine if miRNA levels in aortic valve tissue were related to age.

Methods
To look for a relationship between miRNAs and normal ageing, we examined control aortic valve samples. We calculated Pearson’s or Spearman’s correlation coefficient, the latter used for qPCR results given their non-normal distribution.

Results
Microarray analysis of control samples (n=16) showed no miRNAs with a statistically significant association with age (Pearson’s correlation coefficient, adjusted p-value < 0.05).

Analysing miRNA expression as measured by qPCR in the control aortic valve tissue samples (n=36, age range 22-97 years), there were borderline associations seen with miR-200c-3p and miR-625-5p (Spearman correlation coefficient, unadjusted p-value 0.044 and 0.058 respectively), although visual analysis of the corresponding scatter plots did not provide convincing evidence of a relationship (Supplementary Fig. S4).

Discussion
In healthy aortic valve tissue, there was little effect of age on miRNA expression, with only a borderline association seen with miR-200c-3p and miR-625-5p. There was a wide range of ages in the control samples examined, but the small numbers mean that there may not have been sufficient power to detect smaller associations.

Overall, this analysis suggests that some aortic valve tissue miRNA expression is likely to have a degree of association with age, but this relationship is likely to be small particularly in those validated miRNAs differentially expressed in aortic stenosis .

Supplementary Figure S1. Aortic valve tissue samples shown in a 3-dimensional representation of the principal components of the differentially expressed miRNAs (adjusted p-value < 0.05). Specimens can be seen to cluster together according to presence or absence of aortic stenosis.

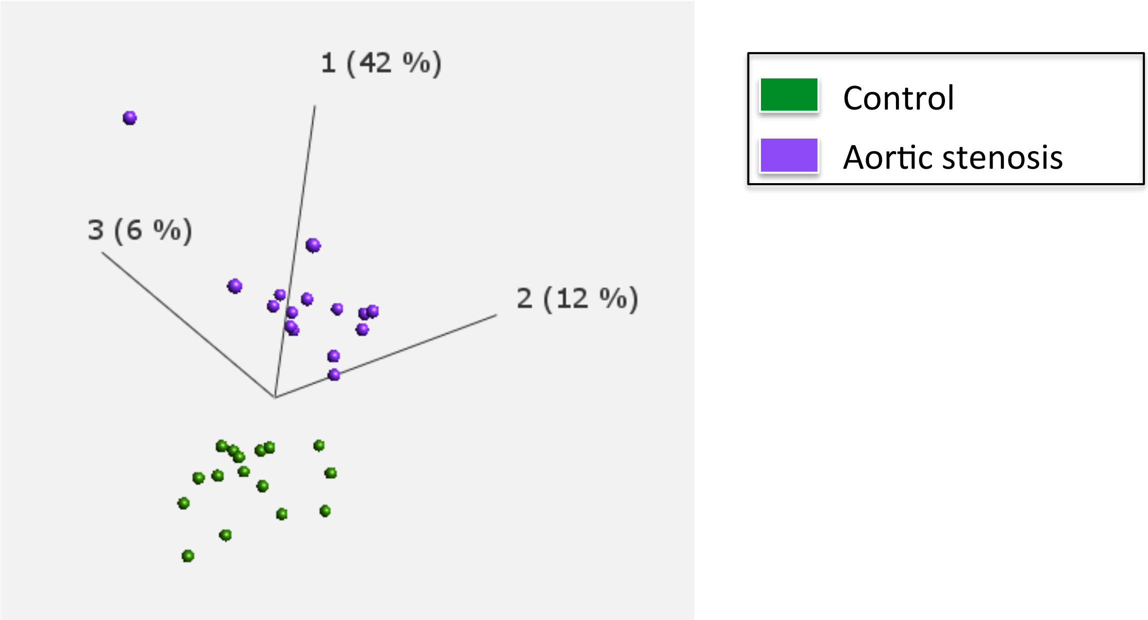

Supplementary Figure S2. Differentially expressed valve tissue related miRNAs (adjusted p-value < 0.05) shown in a 3-dimensional representation of the principal components.

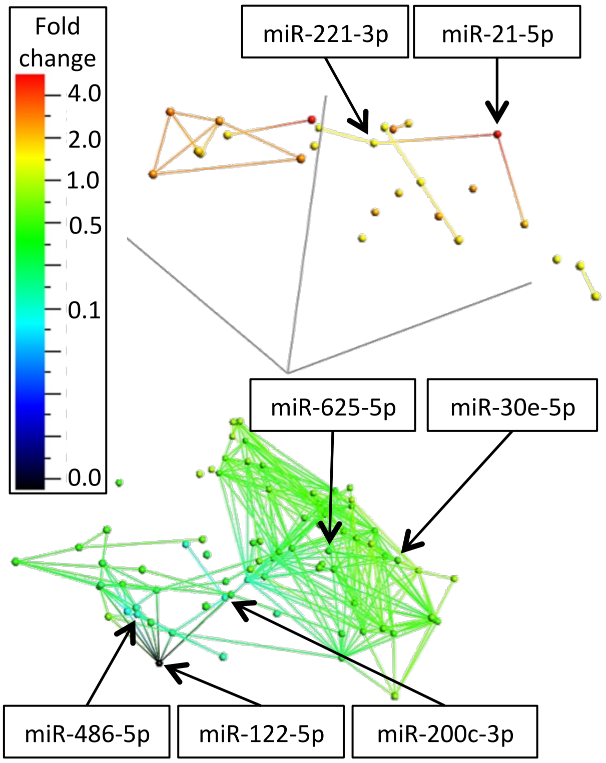

Each node represents a different miRNA, coloured according to fold change. miRNAs subsequently examined by quantitative polymerase chain reaction are labelled. The upper two labelled miRNAs cluster with those with increased expression in severely calcified aortic valve tissue. The lower five are in a large cluster with decreased expression. miRNAs with Pearson correlation ⩾ 70% are linked by a solid line. miR-122-5p is the most differentially expressed miRNA within the decreased expression cluster. Overexpressed miRNAs are less well connected at a correlation of ⩾ 70%, but are all correlated ⩾ 50%.

Supplementary Figure S3. Example of a dissection of a valve leaflet taken from an 86-year-old male participant with aortic stenosis undergoing aortic valve replacement. The portion of the specimen used for analysis was the area of most severe disease.

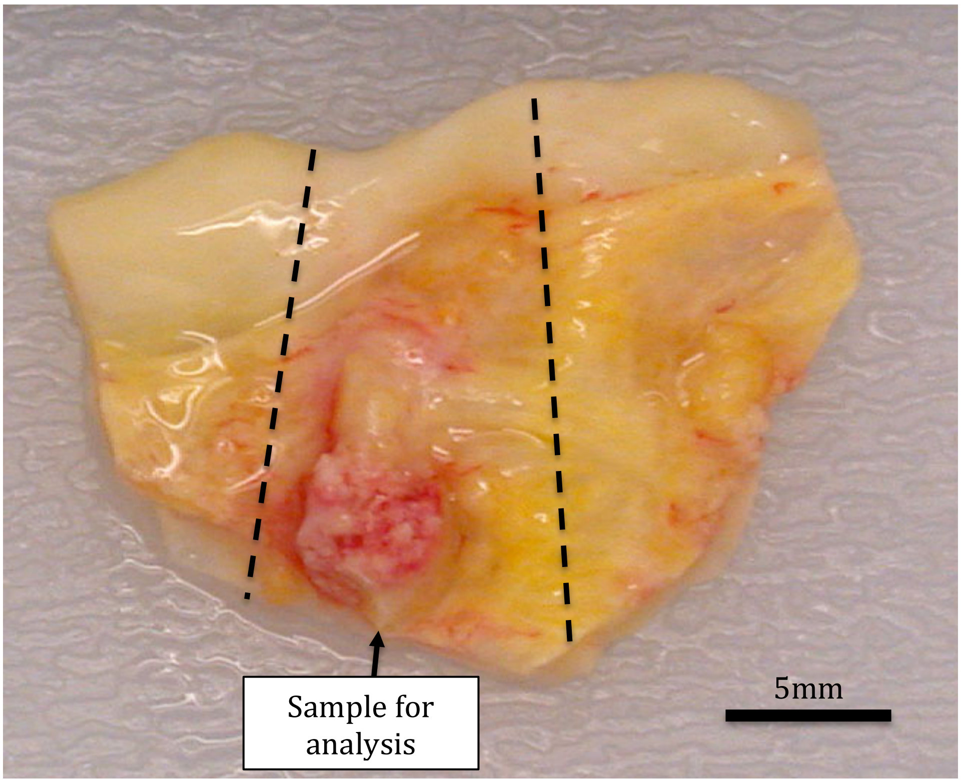

Supplementary Figure S4. Comparison between age of subject and miRNA levels in aortic valve tissue from control participants, as measured by qPCR (n=36). The p-value listed is for Spearman's correlation coefficient.

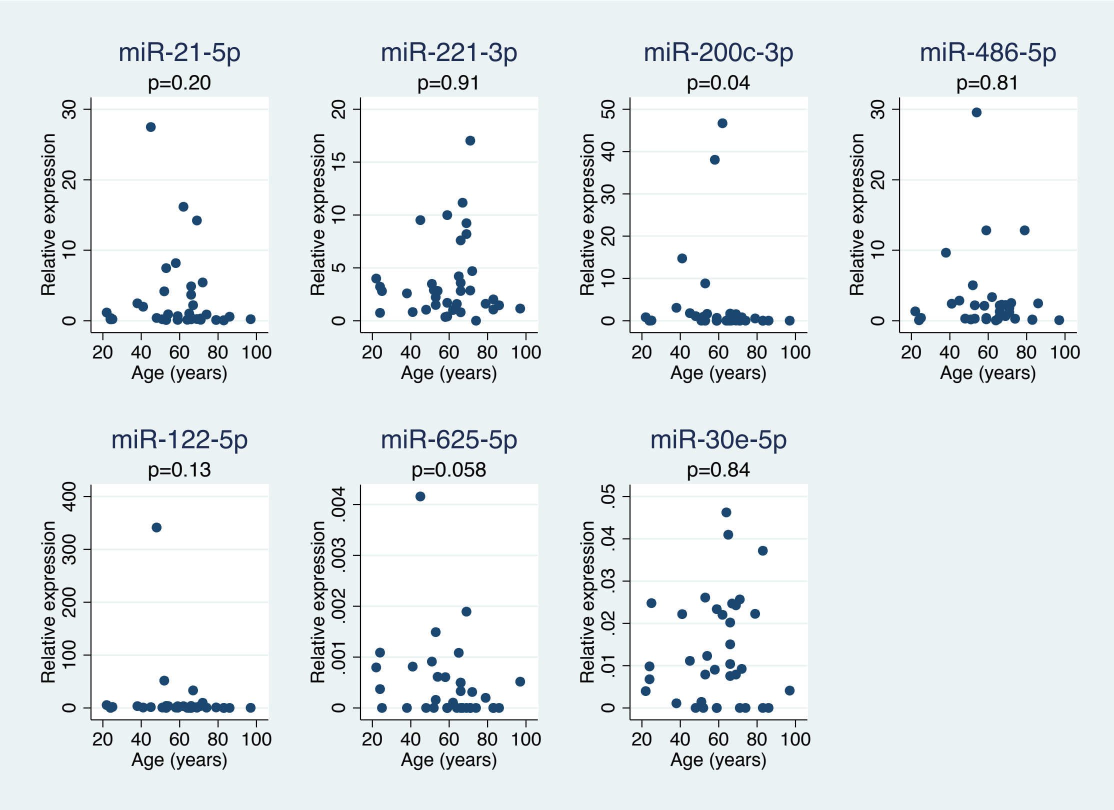

Supplementary Table S1. Differentially expressed miRNAs between aortic valve tissue from patients with aortic stenosis compared to control participants (t-test, adjusted p-value < 0.05).

**Probe Name (Mirbase v20)**	p-value	q-value	Fold change
**Upregulated in aortic stenosis**			
**miR-3197**	1.25E-05	2.52E-04	5.56
**miR-21-5p**	3.44E-08	4.87E-06	5.21
**miR-548a-3p**	2.91E-03	1.14E-02	3.06
**miR-31-5p**	4.24E-04	3.63E-03	3.03
**miR-3128**	1.17E-02	3.32E-02	2.92
**miR-335-5p**	4.38E-04	3.65E-03	2.92
**miR-216a-5p**	6.72E-03	2.16E-02	2.71
**miR-27a-5p**	1.51E-04	1.78E-03	2.67
**miR-1263**	1.54E-03	7.80E-03	2.67
**miR-1275**	2.36E-04	2.56E-03	2.33
**miR-146b-5p**	9.86E-04	5.47E-03	2.21
**miR-3124-5p**	1.87E-03	8.60E-03	2.01
**miR-34a-5p**	1.66E-04	1.88E-03	1.93
**miR-381-3p**	1.12E-02	3.28E-02	1.89
**let-7f-5p**	5.22E-03	1.82E-02	1.80
**miR-3178**	3.06E-03	1.17E-02	1.80
**miR-550a-3p**	1.55E-02	4.15E-02	1.77
**miR-455-3p**	1.16E-03	6.09E-03	1.70
**miR-143-3p**	1.17E-02	3.32E-02	1.56
**let-7i-5p**	8.52E-04	5.32E-03	1.44
**miR-221-3p**	3.38E-04	3.19E-03	1.41
**miR-222-3p**	1.71E-03	8.22E-03	1.39
**miR-199a-3p**	1.99E-03	8.66E-03	1.36
**miR-199b-3p**	9.44E-04	5.47E-03	1.35
**miR-199a-5p**	2.90E-03	1.14E-02	1.35
**miR-27b-3p**	2.94E-03	1.14E-02	1.33
**Downregulated in aortic stenosis**			
**miR-214-3p**	3.80E-03	1.36E-02	0.84
**miR-103a-3p**	3.30E-03	1.23E-02	0.84
**miR-574-3p**	1.04E-02	3.13E-02	0.83
**miR-107**	1.70E-03	8.22E-03	0.81
**miR-320c**	2.77E-03	1.13E-02	0.79
**miR-320b**	5.97E-03	2.01E-02	0.79
**miR-191-5p**	5.85E-03	1.99E-02	0.79
**miR-320a**	6.27E-03	2.06E-02	0.79
**miR-99a-5p**	1.06E-02	3.13E-02	0.78
**miR-197-3p**	1.03E-02	3.13E-02	0.69

**miR-16-5p**	9.25E-05	1.19E-03	0.67
**miR-92a-3p**	7.83E-08	7.14E-06	0.66
**miR-140-3p**	3.28E-06	1.03E-04	0.65
**miR-185-5p**	3.15E-03	1.19E-02	0.62
**miR-502-3p**	1.58E-03	7.86E-03	0.61
**miR-19b-3p**	1.22E-02	3.41E-02	0.60
**miR-18b-5p**	9.24E-04	5.47E-03	0.60
**miR-501-3p**	3.70E-03	1.34E-02	0.60
**miR-629-5p**	1.40E-02	3.81E-02	0.59
**miR-766-3p**	7.29E-04	5.06E-03	0.59
**miR-500a-3p**	3.76E-04	3.44E-03	0.58
**miR-20a-5p**	1.98E-03	8.66E-03	0.58
**miR-30d-5p**	3.61E-03	1.33E-02	0.57
**miR-1225-5p**	1.33E-02	3.66E-02	0.57
**miR-652-3p**	7.39E-03	2.34E-02	0.56
**miR-1287-5p**	7.28E-04	5.06E-03	0.56
**miR-30e-5p**	1.30E-02	3.60E-02	0.54
**miR-128-3p**	2.93E-03	1.14E-02	0.54
**miR-339-5p**	9.03E-03	2.78E-02	0.52
**miR-3162-5p**	1.06E-02	3.13E-02	0.51
**miR-4253**	6.21E-03	2.06E-02	0.51
**miR-149-5p**	1.87E-03	8.60E-03	0.50
**miR-425-5p**	9.55E-04	5.47E-03	0.49
**miR-93-5p**	9.89E-05	1.22E-03	0.48
**miR-324-3p**	1.16E-02	3.32E-02	0.48
**miR-532-5p**	4.87E-03	1.72E-02	0.48
**miR-532-3p**	5.02E-04	4.06E-03	0.48
**miR-17-5p**	1.68E-05	3.17E-04	0.48
**miR-491-5p**	1.40E-03	7.19E-03	0.47
**miR-106b-3p**	7.65E-03	2.38E-02	0.47
**miR-1180-3p**	2.76E-03	1.13E-02	0.47
**miR-18a-5p**	5.27E-03	1.82E-02	0.46
**miR-939-5p**	7.80E-04	5.21E-03	0.46
**miR-885-5p**	1.41E-02	3.81E-02	0.45
**miR-664-5p**	1.95E-03	8.66E-03	0.45
**miR-2110**	3.01E-04	2.97E-03	0.44
**miR-500a-5p**	6.46E-04	4.94E-03	0.43
**miR-1271-5p**	2.12E-03	9.11E-03	0.43
**miR-125b-2-3p**	5.82E-04	4.57E-03	0.43
**miR-665**	7.18E-04	5.06E-03	0.42
**miR-181a-2-3p**	7.44E-03	2.34E-02	0.42
**miR-1207-5p**	9.66E-04	5.47E-03	0.42
**miR-106a-5p**	6.45E-03	2.10E-02	0.40
**miR-328-3p**	8.99E-05	1.19E-03	0.40
**miR-182-5p**	1.88E-03	8.60E-03	0.36

**miR-625-5p**	8.64E-04	5.32E-03	0.35
**miR-422a**	2.53E-04	2.66E-03	0.34
**miR-194-5p**	2.24E-03	9.45E-03	0.33
**miR-378a-5p**	5.92E-06	1.52E-04	0.33
**miR-339-3p**	7.95E-04	5.21E-03	0.32
**miR-1202**	3.95E-04	3.49E-03	0.31
**miR-4284**	3.04E-04	2.97E-03	0.31
**miR-192-5p**	8.10E-04	5.21E-03	0.30
**miR-1**	9.93E-06	2.16E-04	0.29
**miR-20b-5p**	5.35E-06	1.52E-04	0.28
**miR-933**	6.02E-05	8.85E-04	0.27
**miR-1973**	1.01E-03	5.50E-03	0.26
**miR-1246**	1.08E-03	5.76E-03	0.24
**miR-378a-3p**	2.84E-05	5.02E-04	0.23
**miR-486-3p**	1.72E-07	9.76E-06	0.21
**miR-124-3p**	7.32E-04	5.06E-03	0.21
**miR-139-5p**	4.71E-05	7.40E-04	0.20
**miR-133b**	6.26E-05	8.85E-04	0.18
**miR-451a**	1.97E-06	6.95E-05	0.18
**miR-378c**	1.01E-07	7.14E-06	0.16
**miR-486-5p**	2.19E-07	1.03E-05	0.14
**miR-206**	3.66E-05	6.09E-04	0.12
**miR-200c-3p**	1.56E-06	6.30E-05	0.11
**miR-133a-3p**	9.39E-06	2.16E-04	0.10
**miR-122-5p**	1.05E-10	2.96E-08	0.01
